# Supplementary material for: The Influence of Histologic Inflammation on the Improvement of Liver Stiffness Values Over 1 and 3 Years
Source: J Clin Med. 2019 Nov 24;8(12):2065. doi: 10.3390/jcm8122065 (PMC6947085; doi:10.3390/jcm8122065)
Supplement: Supplementary file 1 [file jcm-08-02065-s001.pdf]

Supplementary Material

Supplementary Table S1. Univariable and Multivariable Linear Regression Analyses for Liver Stiffness Measurement in Transient Elastography with Alanine Aminotransferase Lower than 40 IU/L

Variable	Univariable		Multivariable	
	β (95% CI)	*P* value	β (95% CI)	*P* value
Age, *y*	0.117 (-0.041 to 0.274)	.145		
Sex, male	-1.399 (-4.607 to 1.809)	.391		
Viral etiology	-14.129 (-19.056 to -9.201)	<.001	-10.402 (-14.367 to -6.437)	<.001
BMI, *kg/m*^{2}	-0.011 (-0.615 to 0.593)	.971		
Laboratory findings				
Platelet, *10*^{9}*/L*	-0.075 (-0.097 to -0.053)	<.001	-0.036 (-0.055 to -0.016)	<.001
AST, *U/L*	0.295 (0.213 to 0.377)	<.001		
ALT, *U/L*	-0.156 (-0.332 to 0.020)	.083		
Total bilirubin, *mg/dL*	7.523 (5.990 to 9.057)	<.001	4.080 (2.844 to 5.316)	<.001
Albumin, *mg/dL*	-13.684 (-16.287 to -11.082)	<.001	-4.967 (-7.464 to -2.469)	<.001
Prothrombin time, *INR*	20.109 (13.124 to 27.094)	<.001	6.082 (0.431 to 11.733)	.035
Creatinine, *mg/dL*	-0.421 (-1.566 to 0.724)	.470		
Sodium, *mEq/L*	-1.807 (-2.449 to -1.166)	<.001	-0.670 (-1.138 to -0.202)	.005
Liver biopsy				
Fibrosis				
F0	1.000 (reference)		1.000 (reference)	
F1	-1.609 (-22.490 to 19.272)	.879	18.337 (2.946 to 33.728)	.020
F2	0.028 (-20.833 to 20.889)	.998	16.384 (1.018 to 31.750)	.037
F3	4.086 (-16.740 to 24.912)	.699	17.163 (1.890 to 32.437)	.028
F4	14.371 (-6.394 to 35.136)	.174	23.592 (8.348 to 38.837)	.003
Steatosis				
No steatosis	1.000 (reference)			
Mild	2.911 (-0.854 to 6.676)	.129		
Moderate	5.610 (-2.661 to 13.881)	.183		
Severe	1.579 (-10.613 to 13.771)	.799		
Inflammation				
No inflammation	1.000 (reference)		1.000 (reference)	
Mild	2.121 (-4.613 to 8.856)	.535	3.162 (-1.192-7.517)	.154
Moderate	7.422 (0.437 to 14.407)	.037	5.281 (0.738-9.823)	.023
Severe	5.850 (-3.199 to 14.899)	.204	5.616 (-0.477-11.710)	.071

BMI, body mass index; AST, aspartate aminotransferase; ALT, alanine aminotransferase; INR, international normalized ratio; CI, confidence interval.

Supplementary Table S2. Linear regression analysis for 1-year liver stiffness value change amount (sensitivity analysis)

Variable	Univariable		Multivariable	
	**β (95% CI)**	***P* value**	**β (95% CI)**	***P* value**
Age, *y*	-0.013 (-0.075, 0.049)	0.680		
Sex, male	-1.545 (-3.051, -0.039)	0.044		
Viral etiology	2.553 (0.168, 4.938)	0.036		
BMI, *kg/m²*	0.016 (-0.206, 0.238)	0.886		
Laboratory findings				
Platelet, *10⁹/L*	0.04 (0.028, 0.051)	<0.001	0.037 (0.027, 0.047)	<0.001
AST, *U/L*	-0.014 (-0.018, -0.009)	<0.001		
ALT, *U/L*	-0.007 (-0.011, -0.004)	<0.001		
Total bilirubin, *mg/mL*	-2.225 (-2.734, -1.716)	<0.001	-2.062 (-2.524, -1.600)	<0.001
Albumin, *mg/dL*	7.101 (5.768, 8.434)	<0.001		
Prothrombin time, *INR*	-6.58 (-8.164, -4.995)	<0.001	-5.305 (-6.729, -3.882)	<0.001
Creatinine, *mg/dL*	3.260 (2.493, 4.027)	<0.001		
Sodium, *mEq/L*	0.561 (0.268, 0.854)	<0.001		
Liver biopsy				
Fibrosis				
F0	1 (reference)			
F1	5.956 (0.414, 11.498)	0.035		
F2	5.809 (0.358, 11.26)	0.037		
F3	1.375 (-4.005, 6.754)	0.616		
F4	-1.36 (-6.694, 3.974)	0.617		
Steatosis				
No steatosis	1 (reference)			
Mild	-0.767 (-2.436, 0.902)	0.367		
Moderate	1.876 (-1.147, 4.899)	0.224		
Severe	-3.616 (-8.472, 1.24)	0.144		
Inflammation				
No inflammation	1 (reference)		1 (reference)	
Mild	-1.967 (-5.717, 1.784)	0.304	-0.734 (-4.056, 2.588)	0.665
Moderate	-4.191 (-7.941, -0.441)	0.029	-2.104 (-5.434, 1.226)	0.215
Severe	-10.205 (-14.282, -6.127)	<0.001	-7.671 (-11.289, -4.052)	<0.001

Supplementary Table S3. Linear regression analysis for 3-year liver stiffness value change amount (sensitivity analysis)

Variable	Univariable		Multivariable	
	**β (95% CI)**	***P* value**	**β (95% CI)**	***P* value**
Age, *y*	-0.065 (-0.134, 0.004)	0.063		
Sex, male	-2.42 (-4.103, -0.737)	0.005		
Viral etiology	-4.921 (-7.577, -2.264)	<0.001		
BMI, *kg/m²*	-0.023 (-0.273, 0.227)	0.857		
Laboratory findings				
Platelet, *10⁹/L*	0.025 (0.012, 0.038)	<0.001		
AST, *U/L*	-0.012 (-0.017, -0.006)	<0.001		
ALT, *U/L*	-0.006 (-0.01, -0.002)	0.002		
Total bilirubin, *mg/mL*	-1.45 (-2.041, -0.859)	<0.001	-0.925 (-1.499--0.351)	0.002
Albumin, *mg/dL*	5.344 (3.784, 6.905)	<0.001	2.839 (1.237-4.441)	0.001
Prothrombin time, *INR*	-6.149 (-7.954, -4.344)	<0.001	-5.001 (-6.742--3.26)	<0.001
Creatinine, *mg/dL*	2.019 (1.13, 2.909)	<0.001		
Sodium, *mEq/L*	0.455 (0.122, 0.787)	0.007		
Liver biopsy				
Fibrosis				
F0	1 (reference)			
F1	0.696 (-5.629, 7.021)	0.829		
F2	-0.847 (-7.068, 5.374)	0.789		
F3	-4.235 (-10.374, 1.904)	0.176		
F4	-6.524 (-12.611, -0.437)	0.036		
Steatosis				
No steatosis	1 (reference)			
Mild	-0.375 (-2.255, 1.504)	0.695		
Moderate	4.286 (0.942, 7.63)	0.012		
Severe	-3.786 (-8.929, 1.357)	0.149		
Inflammation				
No inflammation	1 (reference)		1 (reference)	
Mild	-5.028 (-9.234, -0.823)	0.019	-4.498 (-8.514--0.483)	0.028
Moderate	-7.792 (-11.997, -3.587)	<0.001	-6.24 (-10.298--2.183)	0.003
Severe	-13.422 (-17.995, -8.85)	<0.001	-11.083 (-15.512--6.654)	<0.001

Supplementary Table S4. Characteristics of patients with paired liver biopsy and paired liver stiffness measurement

Sex/Age	etiology	Liver biopsy interval (years)	Liver stiffness (LS) (kPa)		Histologic findings_Pre*		Histologic findings_Post*		Improvement			Brief history
			Pre	Post	Fibrosis (stage)	Inflammation (grade)	Fibrosis (stage)	Inflammation (grade)	LS	Fibrosis	Inflammation	
F/60	HBV	6	17.3	7	4	2	4	1	Yes	No	Yes	taking lamivudine for over 10 years
F/51	HBV	1	8.1	11.2	2	3	3	2	No	No	Yes	No history of antiviral drugs
F/53	HBV	1	5.3	6.1	3	3	2	1	No	Yes	Yes	taking entecavir for over 9 years
M/60	alcohol	1.5	8.6	8.9	2	1	2	1	No	No	No	current drinker
M/60	HBV	7	4.7	4.3	2	1	1	1	Yes	Yes	No	taking entecavir for over 7 years
F/34	HBV	2	6.3	8.6	3	1	3	1	No	No	No	No history of antiviral drugs
M/69	HBV	5	4.4	4.6	3	2	3	1	No	No	Yes	taking adefovir for over 5 years
F/57	HBV	6	5.2	3.7	3	1	2	2	Yes	Yes	No	No history of antiviral drugs
M/75	HBV	2	6.4	8.9	3	1	4	1	No	No	No	taking entecavir for over 10 years
F/62	autoimmune	3	4.8	7.2	1	1	1	2	No	No	No	Stable state without steroid or immunosuppressant
F/50	HBV	1	6.7	7	2	1	1	1	No	Yes	No	No history of antiviral drugs
F/65	HBV	1	22	18	4	3	4	1	Yes	No	Yes	taking tenofovir for over 8 years
M/63	HCV	1	17.3	17.1	4	1	4	1	Yes	No	No	Sustained viralogic response for 7 years after interferon therapy
M/55	HBV	9	10.4	6.7	4	1	3	1	Yes	Yes	No	No history of antiviral drugs
F/56	HBV	2	48	43.5	4	1	4	2	Yes	No	No	taking tenofovir for over 10 years

M/56	HBV	2	11.6	13.9	4	1	4	1	No	No	No	taking entecavir for over 12 years
F/69	HBV	1	21.8	10.1	4	1	3	1	Yes	Yes	No	taking entecavir for over 7 years
M/59	HBV	1	9	7.9	4	1	4	1	Yes	No	No	taking entecavir for over 9 years
M/42	HBV	2	48	9.5	3	3	4	1	Yes	No	Yes	taking tenofovir for over 6 years
M/49	alcohol	1	7.3	10.5	3	3	4	3	No	No	No	current drinker
F/41	alcohol	7	11.9	39.1	3	2	4	2	No	No	No	current drinker
M/59	HCV	1.5	8.4	4.9	2	2	2	1	Yes	No	Yes	Sustained viralogic response for 8 years after interferon therapy
F/63	alcohol	1	20.8	12.5	3	2	3	1	Yes	No	Yes	current drinker
F/31	autoimmune	2	10.5	21.3	0	2	0	3	No	No	No	Stable state without steroid or immunosuppressant

* Fibrosis was staged on a 0 to 4 scale using METAVIR criteria: F0, no fibrosis; F1, portal fibrosis without septa; F2, periportal fibrosis; F3, septal fibrosis; F4, liver cirrhosis. Inflammation was graded as none, mild, moderate, or severe.

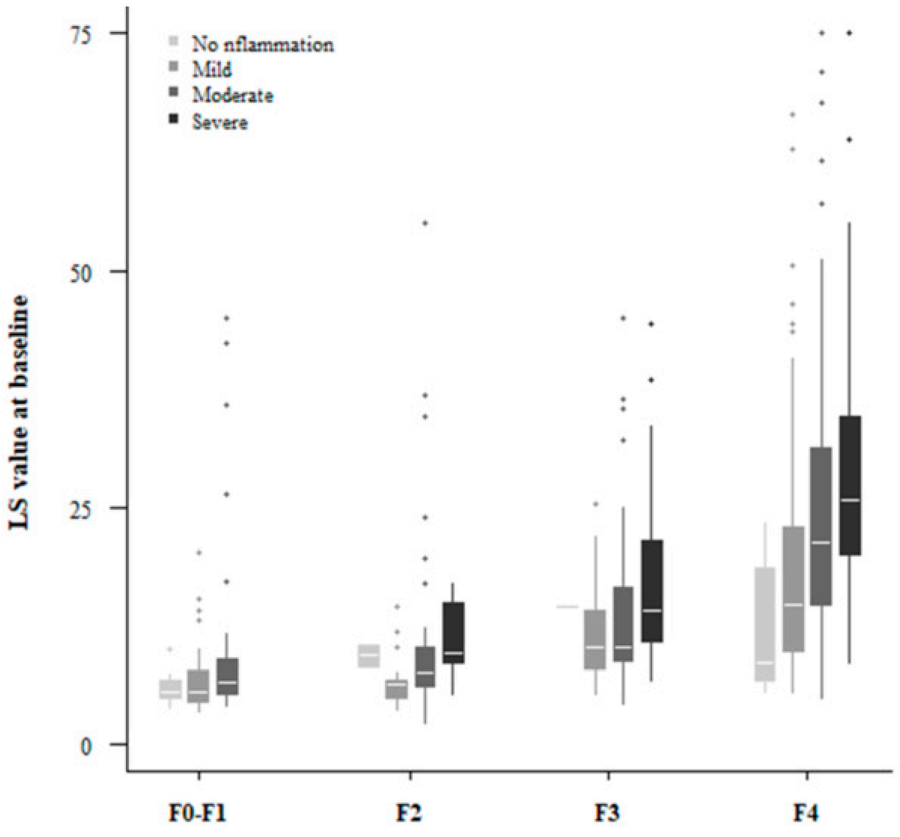

Supplementary Figure S1. Distribution of baseline liver stiffness values according to fibrosis stage and inflammation grade.
